# Differences in the social patterning of active travel between urban and rural populations: findings from a large UK household survey

**DOI:** 10.1007/s00038-014-0578-2

**Published:** 2014-06-26

**Authors:** Jayne Hutchinson, Piran C. L. White, Hilary Graham

**Affiliations:** 1 Department of Health Sciences, University of York, York, YO10 5DD UK; 2 Environment Department, University of York, York, YO10 5DD UK

**Keywords:** Environmental behaviour, Health behaviour, Socio-demographic, Walking, Cycling

## Abstract

**Objectives:**

To determine the social patterning of active travel of short journeys for urban and rural residents in a large UK representative sample.

**Methods:**

Associations between frequently walking or cycling short journeys and socio-demographic factors in the UK Household Longitudinal Study were determined using logistic regression.

**Results:**

Urban residents were 64 % more likely to frequently engage in active travel than rural residents (95 % CI 1.52, 1.77). Being younger, male, without full-time employment and having a lower income independently predicted greater active travel for both urban and rural residents. Degree level education and not having children were independent predictors for urban, but not rural residents.

**Conclusions:**

Actively travelling short journeys is less common and independently associated with fewer socio-demographic factors in rural than in urban populations.

## Introduction

The major risks for population health are seen to lie in individual behaviours, with physical inactivity identified as a risk factor for the non-communicable diseases that account for the majority of deaths in Europe (WHO 2012). However, it is increasingly recognised that changes in the biophysical environment, and increasing global temperatures and ecosystem degradation in particular, constitute even greater threats to long-term population health (Kovats and Butler 2012).

Active travel (AT), defined as walking and cycling for transport, has the potential to produce health and environmental co-benefits. With respect to health, walking and cycling are forms of physical activity associated with lower risks of mortality and reductions in BMI (Andersen et al. 2000; Wagner et al. 2001). Shifting from car to bicycle has been found to deliver health benefits that substantially outweigh the costs associated with greater risks from traffic pollution and road traffic accidents to which cyclists are exposed (de Hartog et al. 2010). With respect to the environment, active travel has low environmental impacts; it produces no particulate matter and no greenhouse gases. In contrast, motorised transport is the major source of transport-related greenhouse gas emissions, dwarfing rail and air transport; the transport sector, in turn, is the largest consumer of energy, exceeding the industrial and service sector (DECC 2013; Eurostat 2013). It is therefore not surprising that AT figures prominently in policies to promote population health and environmental sustainability (Defra 2008; Department of Health 2010; UNECE 2009; WHO 2012). Within Europe, the UK has among the lowest levels of AT; increasing AT is therefore a key policy objective (Defra 2008; Department of Health 2010).

Although AT is high on the policy agenda, understanding of its social patterning remains limited. Furthermore, studies typically focus on particular types of AT (e.g. cycling), travel purposes (e.g. commuting) (Heinen et al. 2009; Laverty et al. 2013) and populations (e.g. urban) (Ogilvie et al. 2008). In population-wide studies, socio-demographic measures can be limited (Goodman 2013), and a distinction between rural and urban populations is typically not included in the analysis (Adams 2010; Kwasniewska et al. 2010). However, UK transport infrastructures and travel patterns, including mode of travel, miles travelled and car ownership, are very different in rural and urban areas (Department of Transport 2013). This suggests that the social profile of AT may be different for urban and rural residents and these populations should be analysed separately.

Here, we use a large UK representative sample from the UK Household Longitudinal Study (UKHLS) to determine the patterning of active travel for urban and rural residents. As far as we are aware, this is the first analysis of the social patterning of active travel among UK adults in a large population survey with rich social data, where account can be taken of rural and urban residents.

## Methods

Over 54,000 adults completed wave 1 of the UKHLS in 2009/2010; this survey is part of Understanding Society (https://www.understandingsociety.ac.uk/) (University of Essex 2013). The analysis excluded 15,000 adults who reported a long-term limiting illness. To account for non-responses and over sampling, the data were weighted incorporating adjustments for both study design and non-responses to produce nationally representative results. This yielded 35,295 weighted individuals for the cross-sectional analyses.

### Measures

Along with a range of socio-demographic measures, the computer-assisted personal interview (CAPI) included the question ‘How often do you personally walk or cycle for short journeys less than 2–3 miles?’ Possible responses were: always; very often; quite often; not very often; never; not applicable, can’t do this. Frequent AT was defined as ‘always’ or ‘very often’ walking and cycling short journeys. ‘Not applicable, can’t do this’ responses (*N* = 627, 1.8 %) were categorised in the analyses as not meeting the outcome, along with the other three remaining responses.

Individual-level socio-demographic measures included age and gender, together with educational attainment (degree, other qualifications, no qualifications) and employment status (full-time, part-time, not employed/retired). Household circumstances were measured by equivalised gross household income split into fifths across the total households (before excluding individuals with long-term limiting illnesses) and household type (no children/children <16 years). Survey participants were categorised by the Understanding Society team as urban if they lived in settlements of 10,000 people or more (as derived from the office of National Statistics Rural and Urban Classification of Output Area); otherwise they were classed as rural.

### Analyses

Chi-squared tests were used to determine significant differences in the percentage of frequent AT between socio-demographic categories for the overall population. Multivariate logistic regression models were used to predict the independent influence of the socio-demographic factors separately for urban and rural residents. First, all the socio-demographic variables were included in forward stepwise logistic regression models which excluded STATA survey weights (forward entry at *p* < 0.1, removal at *p* > 0.15). Only those variables with categories significantly associated with AT were then included in the weighted models; all variables remained significant (at *p* < 0.05). Variables excluded by the first step were separately re-introduced; as these remained non-significant they were not included in the final models. Because car ownership could be acting, at least in part, as a proxy for distance from intended destination and the availability of other modes of transport, we also examined the effect of adjustment for having ≥1 car in the household.

## Results

In the UKHLS, 43 % of participants reported they frequently walked or cycled short journeys (21 % always and 22 % very often). Among the fifth (22 %) living in rural areas, 33 % reported frequent AT compared with 46 % of urban residents (Table 1). Urban residents were 64 % more likely to frequently travel actively than rural residents [OR = 1.64 (95 % CI 1.52, 1.77) after adjusting for age]. In total 16 % did not have a car in their household; this was reported by 19 % of urban but only 7 % of rural residents.

**Table 1:** Descriptive characteristics and percentage of frequent active travel for the UK Household Longitudinal Study sample 2009/2010

	% Of full sample	A. Full sample (*N* = 35,295)		B. Urban (*N* = 27,614)		C. Rural (*N* = 7,681)	
		Frequent active travel?	*p* value	Frequent active travel?	*p* value	Frequent active travel?	*p* value
		Yes %		Yes %		Yes %	
Age			<0.001		<0.001		<0.001
16–24	18.0	54		57		40	
25–34	19.0	45		47		33	
35–44	19.7	40		42		30	
45–54	16.8	39		41		31	
55–64	12.8	39		42		32	
65+	13.6	37		40		32	
Gender			0.1		0.6		0.008
Female	49.8	42		45		31	
Male	50.2	43		46		34	
Highest educational qualification			0.5		0.1		0.07
None	12.3	42		44		35	
Other	63.2	43		45		33	
≥Degree	24.5	43		46		30	
Employment activity			<0.001		<0.001		<0.001
Full-time	49.8	37		40		28	
Part-time	16.2	46		49		36	
Not employed	34.1	50		53		37	
Equivalised household income (UKHLS 5ths)			<0.001		<0.001		<0.001
5 Highest	23.7	36		39		27	
4	23.5	39		42		31	
3	20.5	43		45		35	
2	16.5	48		51		38	
1 Lowest	15.8	53		56		38	
Child in household			0.08		0.7		0.05
Children <16	35.4	44		46		34	
No children	64.6	42		45		32	
At least one car in the household			<0.001		<0.001		<0.001
Yes	84.0	37		40		30	
No	16.0	71		72		63	
Urban/rural resident			<0.001				
Urban	78.2	46					
Rural	21.8	33					

Overall, 54 % of adults below the age of 25 reported they frequently travelled actively, whereas about 39 % of those aged between 35 and 64 years of age did so. AT was reported by 50 % of individuals not in employment and 53 % of those in the lowest household income fifth, compared to 37 % of individuals in full-time employment and 36 % in the highest income fifth (Table 1). The corresponding percentages for urban residents were slightly higher (Table 1), but were substantially lower for rural residents: only 28 % of those in full-time employment and 27 % of those in the highest income fifth frequently travelled actively. AT increased slightly with increased educational attainment for both the full and urban sample; however, AT reduced in the rural sample from 35 % for those with no qualifications to 30 % for those with degree qualifications.

Full sample: in the model which mutually adjusted for all socio-demographic factors except car ownership, all were independent predictors (Table 2, col A). The strongest positive predictors were not being in employment compared to full-time employment [OR (95 % CI) = 1.74 (1.63, 1.86)], being an urban rather than rural resident [OR (95 % CI) = 1.61 (1.49, 1.73)], and being in the lowest compared to the highest household income group [OR (95 % CI) = 1.60 (1.45, 1.77)]. After additional adjustment for having no car in the household, not having children in the household became non-significant (Table 3, col A).

**Table 2:** Predictors of frequent active travel in the UK Household Longitudinal Study 2009/2010 split by urban and rural residents (excluding car ownership)

	A. Full sample (*N* = 35,295)	B. Urban residents (*N* = 27,614)	C. Rural residents (*N* = 7,681)
	OR (95 % CI)	OR (95 % CI)	OR (95 % CI)
Age	0.98 (0.98, 0.99)	0.98 (0.98, 0.99)	0.99 (0.99, 0.99)
Gender			
Female	1	1	1
Male	1.15 (1.09, 1.20)	1.11 (1.06, 1.18)	1.29 (1.17, 1.42)
Highest educational qualification			
None	1	1	
Other	1.05 (0.97, 1.14)	1.09 (1.00, 1.19)	
≥Degree	1.28 (1.16, 1.41)	1.36 (1.22, 1.52)	
Employment activity			
Full-time	1	1	1
Part-time	1.47 (1.36, 1.58)	1.45 (1.33, 1.57)	1.56 (1.34, 1.80)
Not employed	1.74 (1.63, 1.86)	1.77 (1.65, 1.91)	1.61 (1.40, 1.86)
Equivalised household income (UKHLS 5ths)			
5 Highest	1	1	1
4	1.13 (1.04, 1.23)	1.13 (1.03, 1.24)	1.13 (0.95, 1.34)
3	1.28 (1.17, 1.39)	1.26 (1.14, 1.40)	1.31 (1.11, 1.54)
2	1.49 (1.36, 1.63)	1.50 (1.36, 1.67)	1.43 (1.19, 1.71)
1 Lowest	1.60 (1.45, 1.77)	1.63 (1.45, 1.82)	1.43 (1.19, 1.74)
Child in household?			
Children <16	1	1	
No children	1.13 (1.07, 1.20)	1.17 (1.09, 1.25)	
Rural/urban			
Rural	1		
Urban	1.61 (1.49, 1.73)

**Table 3:** Predictors of frequent active travel in the UK Household Longitudinal Study 2009/2010 split by urban and rural residents (including car ownership)

	Full sample (*N* = 35,295)	Urban residents (*N* = 27,614)	Rural residents (*N* = 7,681)
	OR (95 % CI)	OR (95 % CI)	OR (95 % CI)
Age	0.98 (0.99, 0.99)	0.99 (0.99, 0.99)	0.99 (0.99, 0.99)
Gender			
Female	1	1	1
Male	1.20 (1.14, 1.26)	1.16 (1.10, 1.23)	1.36 (1.23, 1.50)
Highest educational qualification			
None	1	1	
Other	1.26 (1.16, 1.37)	1.32 (1.21, 1.45)	–
≥Degree	1.49 (1.34, 1.65)	1.58 (1.41, 1.77)	–
Employment activity			
Full-time	1	1	1
Part-time	1.54 (1.43, 1.66)	1.52 (1.40, 1.66)	1.63 (1.34, 1.80)
Not employed	1.66 (1.56, 1.78)	1.69 (1.57, 1.82)	1.54 (1.40, 1.86)
Equivalised household income (UKHLS 5ths)			
5 Highest	1	1	1
4	1.10 (1.01, 1.19)	1.09 (1.00, 1.19)	1.10 (0.93, 1.31)
3	1.17 (1.07, 1.27)	1.14 (1.03, 1.25)	1.25 (1.06, 1.48)
2	1.19 (1.09, 1.31)	1.17 (1.05, 1.30)	1.24 (1.03, 1.49)
1 Lowest	1.11 (1.01, 1.23)	1.10 (0.98, 1.24)	1.08 (0.87, 1.33)
Child in household?			
Children <16			
No children	–	–	–
Rural/urban			
Rural	1		
Urban	1.42 (1.33, 1.55)		
At least one car in the household			
Yes	1	1	1
No	3.71 (3.43, 4.01)	3.67 (3.37, 3.99)	3.90 (3.20, 4.75)

Urban residents: similarly, in the adjusted model of urban residents all socio-demographic factors were independent predictors (Table 2, col B). Frequent AT decreased with increases in age but increased with decreases in household income. As in the full sample, the strongest positive predictors for urban residents were not being in employment [OR (95 % CI) = 1.77 (1.65, 1.91)] and being in the lowest household income group [OR (95 %CI) = 1.63 (1.45, 1.82)]. Working part-time, being male, not having children in the household and being educated to degree level compared with having no qualifications were also positive predictors of AT. Having no car in the household was very strongly and independently associated with AT [OR (95 %CI) = 3.67 (3.37, 3.99)]. When car ownership was added to the model, not having children in the household and some of the equivalised household income categories became non-significant (Table 3, col B).

Rural residents: fewer socio-demographic factors predicted frequent AT in the adjusted model for rural residents (Table 2, col C) than in the model for urban residents. In contrast to the urban population, neither education nor having children in the household were significantly associated with AT. However, younger age, being male, working part-time or not being in employment remained significant positive predictors, as did being in the three lowest income groups compared to the highest fifth. Associations also tended to be weaker; however, being male was a stronger predictor of AT than in the urban population. As for urban residents, not having a car was a strong independent predictor of AT [OR (95 % CI) = 3.90, CI 3.20, 4.75]. After controlling for this, fewer income categories were significantly associated with AT (Table 3, col C).

In addition, we examined whether the urban–rural differences were inflated by AT prevalence and patterns in London. When the urban analyses were rerun excluding London residents, the urban–rural differences remained (data not shown).

## Discussion

Our analysis of frequently walking or cycling short journeys less than 2–3 miles suggests that actively travelling short distances in the UK is associated with lower income (or no car in household), not being in full-time employment, being younger, and lower educational attainment; these findings are in line with a smaller UK study of AT (≥30 min of AT per day) (Adams 2010). Additionally men in the UKHLS were more likely to report they always or very often travelled actively than women.

We add to existing evidence on AT by capturing important differences between the UK’s urban and rural populations. AT of short journeys less than 2–3 miles was less common among rural residents and was less socially patterned. In contrast to the urban population, neither educational level nor children in the household were independent predictors of active travel in the rural population.

The relative lack of amenities within walking distance and the poorer transport infrastructure in the UK’s rural areas are likely to be major explanatory factors. Close access to amenities and good transport links have been found to be associated with greater AT (Ogilvie et al. 2008; Rissel et al. 2012). In a recent British travel survey, 69 % of rural households lived within 6 min of their nearest bus stop, compared with 90 % of households in medium-sized urban areas (Department of Transport 2013). Additionally, the average distance travelled per year for rural residents is nearly double that in metropolitan built-up areas (Department of Transport 2013). Private transport is therefore more of a necessity in rural areas than in urban ones: in the UKHLS, even in low-income rural households, three-quarters had a car, a finding consistent with a UK study which reported that the local areas with the highest levels of commuting by car were all rural areas (Goodman 2013).

The main strengths of the UKHLS are its large and nationally representative sample and its rich socio-demographic data. Prior AT analyses of UK individuals have incorporated fewer socio-demographic factors (Goodman 2013; Ogilvie et al. 2008), have focused on urban sub-populations (Ogilvie et al. 2008; Panter et al. 2013) and comprised much lower numbers of participants. These studies, therefore may be underpowered to detect associations (Panter et al. 2013). Furthermore, the UKHLS question on AT used in this analysis encompassed both commuting and non-commuting journeys. Some studies focus solely on commuting (Laverty et al. 2013; Panter et al. 2013), yet commuting and business trips make up only a small percentage (18 %) of total UK trips (Department of Transport 2013).

Four in ten participants in the UKHLS reported they always or very often walked or cycled journeys less than 2 or 3 miles. Because AT was subjectively reported and therefore would be subject to response bias, it may be over-reported. Compared to time use diaries (Adams 2010), the single UKHLS question provides a relatively limited measure of AT. However, diaries impose a greater burden on participants and, like accelerometers and Global Positioning Systems (GPS), are likely to be too costly for a large survey. Additionally, this UKHLS AT measure includes both walking and cycling and therefore obscures potential differences in their social profiles; a UKHLS analysis of commuters found males were more likely to cycle and females were more likely to walk to work (Laverty et al. 2013). However, the majority of active travellers are walkers; in the UK National Travel Survey, 22 % of all trips were walked and 2 % cycled, and the average trip length for cyclist was just over three miles (Department of Transport 2013).

Active travel can provide both health and environmental benefits, and thus form part of an integrated approach to improving the health of population and ecosystems. Our analysis of a large contemporary UK study suggests that the prevalence and the patterns of AT of short journeys less than 2–3 miles are different in rural and urban communities; AT is less prevalent and independently associated with fewer socio-demographic factors in rural populations. It could be informative to analyse these populations separately when investigating total physical activity and related health outcomes, as well as when focusing on active travel. Similarly, policy initiatives to encourage AT may be enhanced by different approaches in rural and urban areas.
